# EFDepth: A Monocular Depth Estimation Model for Multi-Scale Feature Optimization

**DOI:** 10.3390/s25237379

**Published:** 2025-12-04

**Authors:** Fengchun Liu, Xinying Shao, Chunying Zhang, Liya Wang, Lu Liu, Jing Ren

**Affiliations:** 1 College of Science, North China University of Science and Technology, Tangshan 063210, China; lnobliu@ncst.edu.cn (F.L.); wangliya@ncst.edu.cn (L.W.); liulu_hblg@ncst.edu.cn (L.L.); renjing@ncst.edu.cn (J.R.); 2 College of Qing Gong, North China University of Science and Technology, Tangshan 063210, China; 3 Hebei Engineering Research Center for the Intelligentization of Iron Ore Optimization and Ironmaking Raw Materials Preparation Processes, North China University of Science and Technology, Tangshan 063210, China; 4 Hebei Key Laboratory of Data Science and Application, North China University of Science and Technology, Tangshan 063210, China; 5 The Key Laboratory of Engineering Computing in Tangshan City, North China University of Science and Technology, Tangshan 063210, China; 6 Tangshan Intelligent Industry and Image Processing Technology Innovation Center, North China University of Science and Technology, Tangshan 063210, China; 7 College of Artificial Intelligence, North China University of Science and Technology, Tangshan 063210, China; sxy@stu.ncst.edu.cn

**Keywords:** deep learning, monocular depth estimation, codec structure, feature enhancement, multi-scale features

## Abstract

**Highlights:**

**What are the main findings?**

- The proposed EFDepth model enhances monocular depth estimation accuracy through encoder–decoder collaborative optimization.
- Achieved excellent performance across multiple key metrics on the KITTI dataset.

**What are the implications of the main findings?**

- The model can provide a more accurate depth map foundation for applications such as 3D reconstruction and robot navigation.
- The encoder-decoder collaborative optimization paradigm offers a novel and effective architectural strategy for tackling the feature-context trade-off in dense prediction tasks.

**Abstract:**

To address the accuracy issues in monocular depth estimation caused by insufficient feature extraction and inadequate context modeling, a multi-scale feature optimization model named EFDepth was proposed to improve prediction performance. This framework adopted an encoder–decoder structure: the encoder (EC-Net) was composed of MobileNetV3-E and ETFBlock, and its features were optimized through multi-scale dilated convolution; the decoder (LapFA-Net) combined the Laplacian pyramid and the FMA module to enhance cross-scale feature fusion and output accurate depth maps. Comparative experiments between EFDepth and algorithms including Lite-mono, Hr-depth, and Lapdepth were conducted on the KITTI datasets. The results show that, for the three error metrics—RMSE (Root Mean Square Error), AbsRel (Absolute Relative Error), and SqRel (Squared Relative Error)—EFDepth is 1.623, 0.030, and 0.445 lower than the average values of the comparison algorithms, respectively, and for the three accuracy metrics, it is 0.052, 0.023, and 0.011 higher than the average values of the comparison algorithms, respectively. Experimental results indicate that EFDepth outperforms the comparison methods in most metrics, providing an effective reference for monocular depth estimation and 3D reconstruction of complex scenes.

## 1. Introduction

Monocular Depth Estimation (MDE) refers to the recovery of a scene’s three-dimensional geometric structure from a single two-dimensional image, representing a fundamental challenge in the field of computer vision. The core difficulty of this task stems from its inherent ill-posed nature: the projection process from the three-dimensional world to a two-dimensional image loses geometric constraints such as depth and scale, theoretically allowing a single image to correspond to an infinite number of valid three-dimensional interpretations [1]. Despite this fundamental challenge, MDE demonstrates immense application potential across numerous domains—including autonomous driving and robotic navigation—due to its advantages of requiring only a monocular camera, low cost, and ease of deployment. For instance, in 3D reconstruction, the quality of predicted depth maps directly determines the accuracy and realism of the reconstructed scene [2].

However, this information gap makes depth prediction models highly susceptible to interference from complex environments in practical applications. Factors such as missing surface textures, extreme lighting variations, and complex occlusions severely limit the estimation accuracy and robustness of existing methods. Therefore, overcoming these bottlenecks to systematically enhance the accuracy, detail preservation capability, and adaptability to complex environments of monocular depth models remains a major research challenge that urgently needs to be addressed in this field.

To address these challenges, research methodologies have evolved from traditional approaches to deep learning-based solutions. Early traditional methods, such as Structure from Motion (SFM) [3] and Multi-View Stereo (MVS) [4], often relied on strong assumptions and exhibited limited generalization capabilities in complex scenes. For instance, MVS methods typically infer depth by analyzing pixel differences in image pairs [5]. While primarily binocular approaches, some variants can be applied to monocular images. Geometry-constrained methods leverage scene geometry and optical flow to estimate depth. Common techniques include monocular depth estimation via photometric consistency [6] and depth estimation using adaptive surface normal constraints and geometric context [7]. Most deep learning-based approaches train neural networks using encoder–decoder (ED) architectures to extract image features and progressively reconstruct depth information. Some neural networks or their variants further enhance accuracy through fully supervised, self-supervised, or unsupervised learning. These methods rely on diverse training strategies and geometric constraints to derive depth information. Deep learning excels at handling complex scenes due to its superior feature extraction capabilities and faster inference speeds [8]. Despite the diversity of techniques, improvements based on the ED framework remain a focal point in academia due to their outstanding performance in both accuracy and efficiency.

Within the mainstream ED framework, researchers have optimized encoders and decoders by incorporating CNNs, Transformers, or hybrid models [9]. However, these approaches still exhibit significant limitations in both feature extraction and context modeling. Regarding feature extraction, the primary issue lies in inadequate fusion of multi-scale features across layers, making it difficult to balance local details with global context. Regarding context modeling, the lack of effective filtering for critical scale information causes disconnect between global and local details, making the model susceptible to background noise interference. Although numerous studies have attempted to address these issues, efficient synergistic optimization between feature extraction completeness and context modeling accuracy remains elusive, presenting a key research opportunity.

Methods based on the encoder–decoder framework are the mainstream approach for monocular depth estimation. This framework extracts features through an encoder and reconstructs depth maps via a decoder. However, shortcomings persist in both feature extraction and context modeling: First, deficiencies in feature extraction manifest as insufficient fusion of multi-scale features across layers [10]. For instance, the Lite-Mono model proposed by Zhang et al. [11] integrates CNN and Transformer features through a local-global feature interaction module. However, its fixed receptive field at the base layer, constrained by conventional convolutional dimensions, struggles to fully capture depth information in large-scale scenes. Yang et al. [12] introduced semantic priors using a pre-trained CNN encoder, yet relied on skip connections to transfer cross-layer features without optimizing semantic matching for multi-scale features, resulting in incomplete feature representation in areas like road edges and building corners; Song et al. [13] achieved feature enhancement through knowledge distillation, thereby improving the performance of deep estimation networks. While this enhanced the weighting of local features, it lacked a comprehensive feature interaction mechanism throughout the entire process. The SS-MDE method proposed by Hu et al. [14] optimizes encoder–decoder training by introducing a teacher-student distillation mechanism, enhancing the model’s depth estimation accuracy and robustness. Although this reduced computational complexity, it still exhibited deficiencies in the coherence of multi-scale feature fusion.

On the other hand, inadequate context modeling primarily manifests as the absence of scale filtering and the disconnect between global and local information. Song et al. [15] introduced Laplacian pyramids to optimize decoder gradient flow and enhanced depth continuity through multi-scale feature superposition. However, they failed to effectively distinguish the importance of features across different levels, leading to interference from irrelevant features such as background noise in context modeling. Zhu et al. [16] embedded a Gate Recurrent Unit (GRU) temporal module within the DPT (Dense Prediction Transformer) encoder–decoder framework to leverage temporal information for global context modeling. However, achieving precise matching of local context for high-frequency spatial details remains challenging; The Align3r method proposed by Lu et al. [17] achieves depth estimation by incorporating the DUSt3R (Dense Unconstrained Stereo 3D Reconstruction) model into an encoder–decoder architecture for feature optimization, but it fails to adapt to contextual information under varying lighting conditions. Yang et al. [18] designed a hybrid encoder with an adaptive decoder, enhancing monocular depth estimation performance by strengthening the fusion of local and global information. However, this multi-frame input dependency fails to meet context modeling demands in applications like autonomous driving. Consequently, context discontinuities frequently occur in scenes with alternating light and shadow.

Although previous studies have attempted to address the aforementioned issues, certain limitations persist. Hwang et al. [19] employed a CNN encoder to extract local features and introduced a Transformer into the global self-attention module, enhancing feature interaction capabilities through self-supervised learning. However, they failed to optimize for the differences in receptive fields across multi-scale features. Li et al. [20] proposed the BinsFormer model, which employs a Transformer decoder for cross-scale attention computation to generate deep interval centers. However, it fails to resolve the issue of semantic discontinuity across feature layers. Zhao et al. [21] introduced the MonoViT model, utilizing a dual-branch CNN-ViT architecture for feature extraction combined with a convolutional upsampling decoder for information fusion. Yet, it lacks a dynamic filtering mechanism for critical scale features. Li et al. [22] designed a parallel encoder architecture where a convolutional branch preserves local details while a Transformer branch models long-range dependencies. Although this enhances feature diversity, it remains inefficient in multi-scale feature fusion. Furthermore, Lyu et al. [23] enhanced the ED structure by optimizing skip connections and introducing a feature fusion module. While this improved depth accuracy in high-gradient regions (e.g., cliff edges), it failed to achieve synergistic optimization between feature extraction and context modeling. Godard et al. [24] proposed the Monodepth2 model, which optimizes the ED framework through minimum reprojection loss and full-resolution multi-scale sampling. Although it has become a domain benchmark, there is still room for improvement in feature robustness under complex lighting conditions [8]. Liu et al. [25] proposed the FCMdepth depth estimation framework, which incorporates feature optimization and contextual information. However, improvements in feature extraction and contextual modeling are still needed to achieve more accurate depth estimation. Wang et al.’s [26] MG-Mono achieves improved accuracy in self-supervised monocular depth estimation while maintaining a lightweight design. This is accomplished by introducing a multi-granularity information fusion module in its encoder to integrate pixel, local, and global features, and by employing a neighborhood-weighted collaborative prediction head in its decoder. However, feature extraction is not sufficiently detailed, and contextual information is not comprehensive enough, leaving room for optimization. Some of these methods have not proposed systematic solutions from the perspective of synergistically optimizing feature extraction completeness and context modeling accuracy, while others still have room for improvement in feature optimization and context modeling capabilities.

The aforementioned methods effectively enhance the accuracy of monocular depth estimation, yet there remains room for further optimization in feature extraction and context modeling. Many existing approaches tend to optimize encoders (feature extraction) or decoders (context modeling and reconstruction) independently, failing to fundamentally address the collaborative challenges between them in semantic representation and scale perception. This separate optimization makes it difficult for models to achieve optimal global performance. To this end, we propose a novel monocular depth estimation model, EFDepth, whose main contributions include the following aspects:

- The EC-Net design encoder achieves optimized multi-scale feature extraction. Addressing the limitations of the MobileNetV3 architecture—insufficient shallow-to-deep information fusion and restricted receptive fields in lower layers—we designed the EC-Net encoder, comprising MobileNetV3-E and ETFBlock modules, to enhance cross-layer feature interaction. Hollow convolutions expand receptive fields, providing decoders with both granular and globally contextual feature representations. Experiments were conducted on the public KITTI dataset (Karlsruhe Institute of Technology and Toyota Technological Institute at Chicago);
- The decoder LapFA-Net enhances contextual modeling capabilities. A five-level Laplacian pyramid performs bottom-up upsampling layer by layer. Each layer implements feature skip connections corresponding to the encoder layer. A Fourier Modulated Attention (FMA) mechanism is introduced at the base layer to select critical scale information. Residual L1–L5 cascades refine high-frequency details, ultimately producing a high-quality depth map that combines global consistency with sharp edges;
- EFDepth, a monocular depth estimation model based on a U-Net-like encoder–decoder architecture, achieves refined optimization of multi-scale features by constructing a dedicated encoder EC-Net and decoder LapFA-Net. This effectively enhances information exchange and fusion between upper and lower layers of the model.

The core innovation lies in achieving synergistic effects between feature extraction and depth reconstruction through the collaborative interaction between encoders and decoders. This powerful combination ultimately enhances depth map prediction performance.

The EC-Net encoder focuses on enhancing multi-scale features, extracting rich, multi-scale details to lay the foundation for depth estimation. The LapFA-Net decoder employs the FMA module to recalibrate and filter this feature stream, enabling precise depth map reconstruction. This synergistic mechanism ensures the encoder provides information-rich input to the decoding process through feature enhancement, while the decoder guides more accurate depth map reconstruction via its internal information filtering mechanism. This enhances model performance, ultimately improving depth estimation accuracy in complex scenes.

Experimental results demonstrate that the EFDepth monocular depth estimation model can provide a relatively accurate depth map foundation for applications such as 3D reconstruction.

The overall structure of this paper is as follows:

Section 2. Related Work briefly introduces prior work closely related to this study;

Section 3. EFDepth Model focuses on the proposed EFDepth model, including its overall framework and the design of the encoder EC-Net and decoder LapFA-Net;

Section 4. Experiments and Results Evaluation presents experiments and evaluates results to validate the effectiveness of the FCMdepth model;

Finally, the research findings are summarized, and future directions are explored.

## 2. Related Work

### 2.1. Encoder–Decoder Structure

The Encoder–Decoder (ED) architecture has been widely adopted for monocular depth estimation tasks. The encoder extracts multi-scale features from the input RGB image through convolutional and pooling layers, enhancing its ability to capture both local details and global information [27]. The decoder progressively upscales feature maps to reconstruct depth maps. To enhance feature utilization efficiency, skip connections are typically introduced between the encoder and decoder, enabling the decoder to fuse multi-scale features from various encoder levels. This architecture, characterized by its ability to extract rich features and accurately reconstruct depth maps, has emerged as an effective monocular depth estimation solution due to its simplicity and stable training.

The basic encoder–decoder architecture adopted in this paper draws inspiration from the design concepts presented in [10,11,12,13,14,15,16,17,18]. The encoder employs MobileNetV3 as the feature extraction network. Given MobileNetV3’s efficiency and superiority in feature extraction, it is selected as the foundational encoder. The decoder utilizes a pyramid structure with Laplacian residuals, performing layer-by-layer upsampling to restore depth map details. Laplacian residuals refine feature representations, enhancing the accuracy and detail preservation in depth map reconstruction [28]. Building upon this encoder–decoder architecture, this paper introduces corresponding improvements to achieve higher-quality monocular depth estimation.

### 2.2. MobileNetV3 Network

**Figure 1:**
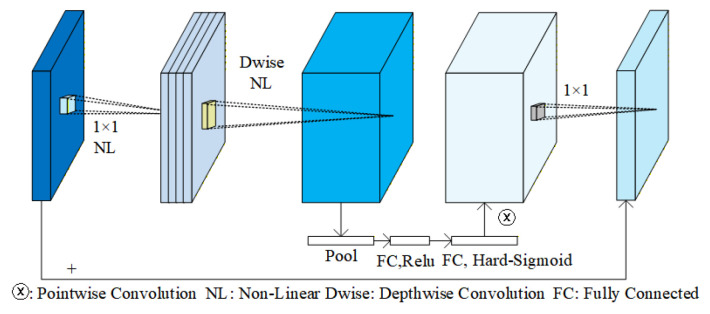
MobileNetV3 Block [30].

MobileNetV3 is an efficient convolutional neural network that employs hardware-aware architecture search and innovative design based on automated machine learning techniques [29]. By automatically optimizing network structure parameters, MobileNetV3 enhances feature extraction capabilities and inference speed while maintaining lightweight architecture [30]. Its core modules (as shown in Figure 1) utilize dilated convolutions, depthwise separable convolutions, and pointwise convolutions to extract multi-scale features. Combined with lightweight attention mechanisms and efficient activation functions, these components significantly boost the network’s expressive power.

MobileNetV3 optimizes the feature extraction process through depthwise separable convolutions, pointwise convolutions, global pooling, fully connected layers, and nonlinear activation functions (ReLU and Hard-Sigmoid) [31]. In this architecture, depthwise separable convolutions significantly reduce the computational load, while pointwise convolutions enhance feature representation. The nonlinear activation functions improve the network’s fitting ability. The fully connected layers generate channel weights, which are used to perform channel-wise weighting, enhancing feature representation and further improving detail recovery and context modeling [32]. This enables MobileNetV3 to effectively improve the accuracy and robustness of monocular depth estimation while maintaining a lightweight design, particularly enhancing adaptability in complex scenes.

MobileNetV3 demonstrates robust feature extraction capabilities in monocular depth estimation [33], particularly in detail recovery and context modeling, thereby improving depth prediction accuracy and robustness.

## 3. EFDepth Model

### 3.1. Overall Framework of the Network Model

**Figure 2:**
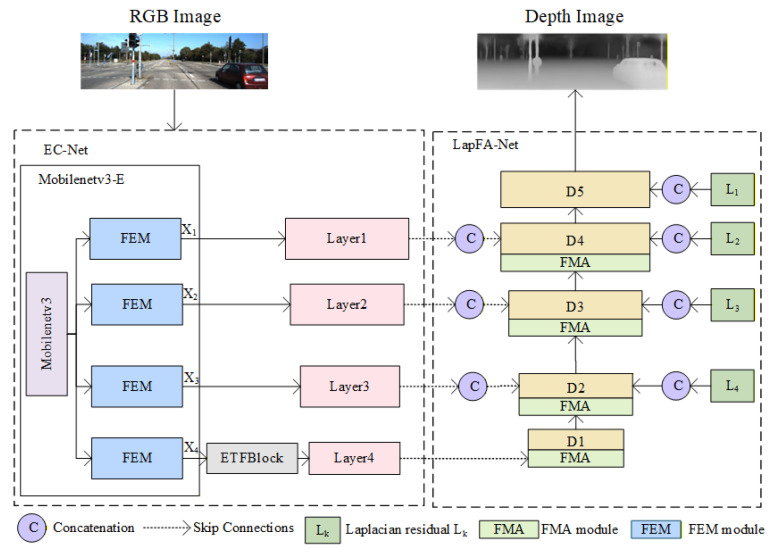
EFDepth network model architecture diagram.

The EFDepth model adopts a U-shaped network encoder–decoder architecture, as shown in Figure 2.

The EC-Net encoder is based on the MobileNetV3 architecture and consists of two modules: ① The MobileNetV3-E module is formed by introducing a Feature Enhancement Module (FEM) into the MobileNetV3 network. This module integrates multi-scale information to enhance feature extraction capabilities, enabling the network to capture richer spatial and semantic features. ② The ETFBlock module refines low-level features by employing dilated convolutions to expand the receptive field, strengthening the expression of granular details and enabling the network to capture broader contextual information.

The decoder LapFA-Net employs a five-level Laplacian pyramid incorporating an FMA module. During upscaling, skip connections and residual fusion with the preceding layer refine the predicted depth map layer by layer. This approach ensures the network maintains both global consistency and high-frequency detail, ultimately reconstructing a high-accuracy depth map.

### 3.2. Encoder EC-Net

EC-Net consists of MobileNetV3-E and ETFBlock modules, which synergistically optimize the feature extraction process. FEM can be employed for feature enhancement. Thus, MobileNetV3-E enhances its multi-scale feature extraction capabilities by introducing the FEM module after MobileNetV3, enabling it to capture rich spatial and semantic information. Meanwhile, the ETFBlock module expands the receptive field through dilated convolutions, allowing the network to better capture long-range dependencies and global contextual information. This collaborative design optimizes feature representation capabilities, thereby improving the features extracted throughout the entire encoding process.

#### 3.2.1. MobileNetV3-E Module

**Figure 3:**
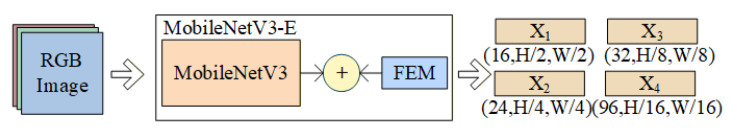
MobileNetV3-E module structure diagram.

Given the performance bottlenecks encountered by MobileNetV3 when processing multi-scale features and fine-grained information, a FEM module was introduced after MobileNetV3 to form the MobileNetV3-E module. This aims to enhance multi-scale feature extraction and feature augmentation capabilities. Its network architecture is illustrated in Figure 3.

After processing through the MobileNetV3-E module, RGB images are extracted into feature maps at four scales (X_1_, X_2_, X_3_, X_4_). Their spatial dimensions are progressively halved: X_1_ is half the size of the original image, X_2_ is one-quarter, X_3_ is one-eighth, and X_4_ is one-sixteenth. Concurrently, the number of feature channels increases progressively. Consequently, X_4_ possesses the smallest spatial scale while carrying the richest channel information and the highest-level semantic details, providing multidimensional support for depth estimation tasks.

In Figure 3, the four feature maps (X_1_–X_4_) from the MobileNetV3-E module represent feature maps at different scales. Within the MobileNetV3-E module, the FEM is inserted after each feature map extracted by MobileNetV3. The features extracted by MobileNetV3 are then processed by the FEM, enhancing multi-scale feature extraction capabilities and improving the perception of both local and global information.

**Figure 4:**
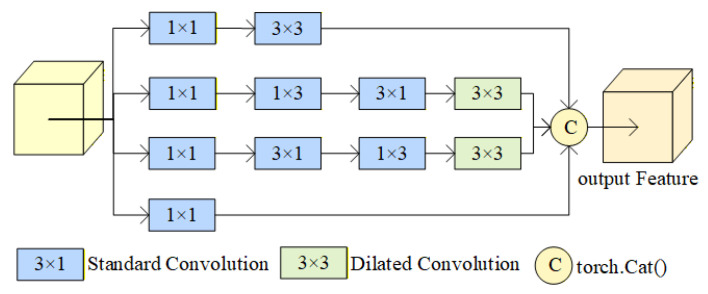
FEM structure diagram [30].

The FEM in MobileNetV3-E (as shown in Figure 4) originates from the FFCA-YOLO model [34], initially designed to enhance detection capabilities for small objects. In FFCA-YOLO, the FEM enriches feature diversity through a multi-branch dilated convolution architecture, expanding the model’s local perception capabilities to enhance semantic information representation for small objects. The introduction of the FEM addresses MobileNetV3’s limitations in detail perception, enabling it to better handle small objects in complex scenes, enrich local feature information, and improve the model’s ability to capture fine-grained features, ultimately achieving feature enhancement.

The FEM first performs preliminary processing on input features through multiple 1 × 1 convolutional layers. Subsequently, it extracts features at different scales in parallel using standard convolutions and dilated convolutions. Finally, the extracted feature maps are concatenated using torch.cat(). This enables the model to simultaneously capture both local and global information. By employing 3 × 3 dilated convolutions to expand the receptive field, the model’s ability to model multi-scale features is enhanced, improving feature extraction and depth estimation capabilities in complex scenes. This fusion of local and global information improves the model’s ability to capture fine-grained features. Building upon multi-scale features, it enhances the expression of semantic information, elevating the model’s overall representational capability. This enables more precise capture of detailed information in complex scenes, achieving feature enhancement.

After processing RGB images through the MobileNetV3-E backbone, four feature maps are generated sequentially, each possessing distinct spatial resolutions and channel dimensions. These form complementary representations at both scale and semantic levels, capturing both fine-grained and global scene information.

#### 3.2.2. ETFBlock Module

**Figure 5:**
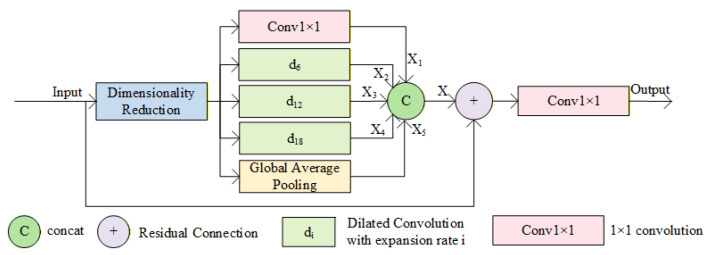
ETFBlock module structure diagram.

After feature enhancement via MobileNetV3-E, the lowest-level features in the encoder retain rich local details and texture information but suffer from significantly reduced spatial resolution. This results in limited feature receptive fields, making it difficult to effectively capture long-range dependencies and global scene structures within images. This lack of global contextual information deprives the decoder of sufficient scene-level semantic guidance during subsequent upsampling and depth map prediction. Consequently, the overall consistency of depth estimation is compromised, leading to prediction errors and structural distortions. To mitigate this issue, drawing inspiration from Atrous Spatial Pyramid Pooling (ASPP) [35], the ETFBlock module (structural diagram shown in Figure 5) is designed to capture multi-scale contextual information, expand the receptive field, enhance the model’s contextual modeling capability, and achieve feature optimization.

Traditional ASPP achieves multi-scale perception and effectively expands the receptive field by fusing convolutional features with different expansion rates. However, in monocular depth estimation tasks, the balanced fusion of global and local information remains an unsolved challenge. Building upon ASPP’s multiscale perception advantages, ETFBlock introduces innovative designs to achieve precise fusion of global-local information: it optimizes feature representation through multiscale context awareness and feature reuse mechanisms. Compared to ASPP, it enhances the model’s adaptability to complex scenes in prediction tasks. The dimension reduction layer serves as a preprocessing step, employing 1 × 1 convolutions to perform channel compression on input features, thereby reducing the number of parameters for subsequent operations. To comprehensively capture multi-scale contextual information, this module deploys multiple feature extraction paths in parallel: 1 × 1 convolutions preserve core features at the original scale while performing linear transformations; Three sets of dilated convolutions with varying dilation rates progressively expand receptive fields, precisely extracting hierarchical contextual information from local details to global structures; a global mean pooling layer generates image-level global feature descriptors by compressing spatial dimensions, infusing scene-level prior knowledge into the model. Subsequently, by sequentially fusing feature maps from each path, it achieves complementary integration of local details, multi-scale context, and global information. ETFBlock introduces residual connections, overlaying the original input features onto the concatenated multi-scale features. This enables the model to focus on residual learning between target features and input features, simplifying the optimization objective while ensuring the transmission of all feature information. Finally, 1 × 1 convolutions perform channel adjustment and cross-channel information integration on the fused and enhanced features, generating optimized output features. This hierarchical collaborative design enhances ETFBlock’s feature richness, with the feature fusion process within the ETFBlock module described by Equation (1).

(1) $$X=concat\left(X_{1},X_{2},X_{3},X_{4},X_{5},\right)$$

In Equation (1), X represents the merged features, and $X_{i}$ denotes the features to be concatenated.

The ETFBlock module achieves multi-scale feature optimization through collaboration with the MobileNetV3-E module, expanding the receptive field and enhancing contextual modeling capabilities. By capturing both local and global information through multi-scale perception, and further integrating residual connections with low-level features, ETFBlock significantly boosts feature expressiveness. This synergistic design ensures a balanced trade-off between detail and global consistency in depth estimation, thereby improving performance in complex scenes.

The multi-scale feature maps (X_1_–X_4_) extracted by MobileNetV3-E are renamed for subsequent reference: X_1_, X_2_, and X_3_ are sequentially designated as Layer1, Layer2, and Layer3. X_4_ requires further processing through the ETFBlock, and its output is named Layer4.

### 3.3. Decoder LapFA-Net

The decoder of LapFA-Net adopts a five-layer Laplacian pyramid architecture. It optimizes multi-scale features through the encoder and progressively restores the spatial resolution of the depth map layer by layer during decoding. To further enhance detail reconstruction quality, the network incorporates a FMA. This module extracts frequency-domain information and performs feature selection, thereby strengthening the decoder’s ability to model local structural features. Simultaneously, the FMA module integrates with multi-scale feature fusion to further strengthen the model’s contextual modeling performance. At each decoder layer, synergistic interaction with the FMA module not only achieves high-quality detail recovery in depth maps but also ensures the consistency and accuracy of depth estimation results across the entire scene.

**Figure 6:**
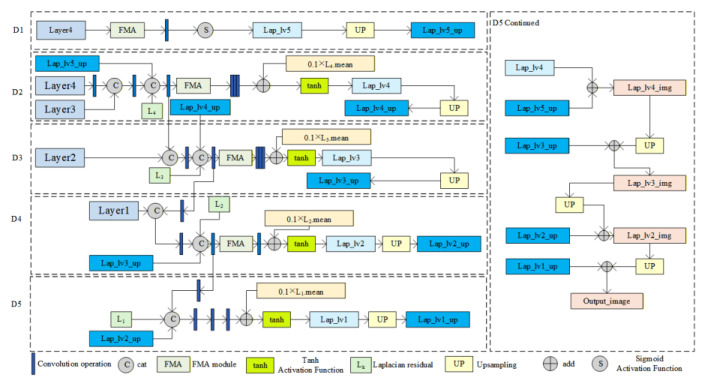
Decoder LapFA-Net structure diagram.

LapFA-Net (as shown in Figure 6) employs a five-layer Laplacian pyramid decoder. During each upsampling step, it utilizes skip connections to incorporate encoder features while introducing Laplacian residuals. Concurrently, it employs the FMA module [36] to progressively refine depth map predictions. The decoder achieves high-quality depth estimation by extracting multi-scale features from different encoder layers (Layer4 to Layer1), progressively restoring the depth map’s spatial resolution layer by layer while refining depth details. The decoding process begins with low-resolution, high-semantic features from Layer4. Through a series of upsampling operations, it gradually enlarges the feature map dimensions and fuses them with corresponding encoder layer features. This integration of global semantic information and local details enhances the accuracy of depth estimation.

The LapFA-Net decoder employs a Laplacian pyramid architecture to predict depth residuals at multiple scales. Lower-resolution scales primarily handle overall depth trend prediction, while higher-resolution scales focus on detail recovery. Through the progressive accumulation of multi-layer residuals, the model ultimately generates detailed and accurate depth maps. Each layer reintroduces the mean-scaled Laplacian residual, progressively balancing feature scales across layers. This enhances model stability, mitigates gradient vanishing issues, and improves training efficiency. Furthermore, by employing diverse nonlinear activation functions, the model effectively captures complex nonlinear mapping relationships. This ensures depth predictions align more closely with real-world depth variation patterns.

Laplacian residuals introduce edge features to assist the decoder in refining and reconstructing images, enabling progressive refinement of depth map predictions [37]. The Laplacian residual calculation formula is given by Equation (2).

(2) $$L_{k}=I_{k}-Upsampling\left(I_{k+1}\right),\;k=0,\;1,\;2,\;3,\;4,\;5$$

In Equation (2), $I_{k}$ denotes the result after subsampling the original image, $I_{0}$ represents the original image, and $Upsampling\left(a\right)$ indicates bilinear upscaling of a. $L_{k}$ is obtained by subtracting the bilinearly upscaled $I_{k}$ from $I_{k+1}$, where $L_{k}$ represents the computed Laplacian residual.

The FMA module originates from the SRConvNet architecture. By integrating Fourier transforms with spatial domain feature interactions, it replaces traditional computations with linear-complexity frequency domain operations while partitioning local windows. This approach reduces computational and parameter overhead while simultaneously capturing both long-range and short-range dependencies to suit lightweight tasks. It also suppresses irrelevant feature responses and enhances texture edge details, enabling high-quality image reconstruction with efficient global information capture capabilities [36]. Given the critical importance of global information in depth prediction within depth estimation model decoders, the FMA module is introduced into the LapFA-Net decoder to enhance the structural consistency and texture detail restoration accuracy of depth maps.

The FMA module is only introduced in decoder layers D1 to D4 and is not used in layer D5. The application of the FMA module in the decoder is based on a grouping mechanism. Given the limited number of channels in the D5 layer, introducing this module may not only incur unnecessary computational overhead but also lead to excessive feature smoothing. This could result in the loss of high-frequency details and compromise depth map accuracy. Furthermore, the core task of the D5 layer is to generate the final depth map. At this stage, features have undergone sufficient fusion and refinement, necessitating the preservation of more features to ensure detail recovery capability.

Decoder layers D1 to D4 primarily handle the fusion and refinement of multi-scale features. The FMA module effectively enhances the expressive power of medium-to-low resolution features, thereby improving detail recovery accuracy. Incorporating 0.1 × Li.mean into each layer compensates for potentially lost detail information and increases feature diversity, further boosting the model’s detail recovery capability. The FMA module filters key information from multi-scale features, preserving depth map details while ensuring overall structural integrity. Ultimately, this enhances depth map prediction accuracy and detail recovery capabilities.

**Figure 7:**
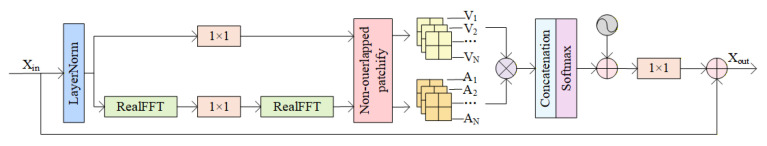
FMA module structure diagram [36].

To process information at different scales more efficiently, enhance focus on detailed regions, and reduce occlusion and blurring, the decoder incorporates FMA modules in its first four layers (as shown in Figure 7). The FMA module transforms input feature maps from the temporal domain to the frequency domain via Fourier transform, capturing frequency information across different scales. It then divides the feature maps into multiple regions using non-overlapping block segmentation and assigns weights to each region through an attention mechanism, thereby amplifying features in critical areas. After attention-weighted feature concatenation, the final output is obtained through deep processing involving Softmax and 1 × 1 convolutions. By extracting frequency information and implementing adaptive feature selection, the FMA module enhances the model’s ability to focus on local details. This optimizes depth map detail recovery while improving feature selection efficiency. Combined with a pyramid structure, it enhances contextual modeling capabilities, emphasizing both local details and global context. Ultimately, this achieves more accurate and refined depth predictions.

The LapFA-Net decoder integrates global semantic information and local details through the fusion of multi-scale features, a Laplacian pyramid structure, precise application of the FMA module, and residual learning, ultimately generating high-accuracy depth maps.

## 4. Experiments and Results Evaluation

### 4.1. Experimental Environment

In the experiment, the EFDepth neural network model was constructed using the PyTorch framework and Python language. Model debugging and training were conducted on a Windows 11 system. Training hardware comprised an NVIDIA GeForce RTX 3060 Laptop GPU with 8GB VRAM, leveraging PyTorch 2.0.0 accelerated by CUDA 11.8. Python version 3.9 was employed. Following training, model evaluation was performed on Ubuntu 20.04 running on identical hardware specifications.

### 4.2. Dataset

**Figure 8:**
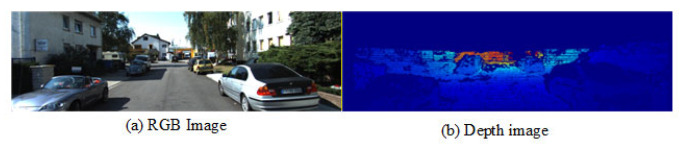
KITTI datasets sample image display.

The KITTI dataset (as shown in Figure 8) is a widely used dataset in the field of computer vision, providing images with corresponding lidar point cloud data. Its 64-line lidar generates high-resolution 3D point clouds that can be aligned with RGB images using camera parameters, yielding sparse depth maps (commonly used as ground truth for depth estimation). The NYU Depth V2 dataset features dense depth maps alongside their corresponding RGB images, enabling training, evaluation and testing. The training process employs a fully supervised strategy, with labels being either lidar depth maps or dense depth maps.

### 4.3. Evaluation Indicators

The evaluation metrics align with previous literature [6,7,8,9,10,11,12,13,14,15], employing Root Mean Square Error (RMSE), Root Mean Square Logarithmic Error (RMSELog), Absolute Relative Error (AbsRel), Square Relative Error (SqRel), and accuracy (δ). Calculation methods are detailed in Equations (3)–(7).

(3) $$RMSE=\sqrt{\frac{1}{\left|T\right|}\sum_{\hat{d}\in T}\frac{{\left\|\hat{d}-d\right\|}^{2}}{d}}$$

(4) $$AbsRel=\frac{1}{\left|T\right|}\sum_{\hat{d}\in T}\frac{\left|\hat{d}-d\right|}{d}$$

In Equations (3) and (4), T denotes the total number of samples in the test set, i.e., the number of pixels in the actual depth map; d represents the actual depth value, while $\hat{d}$ denotes the depth value predicted by the model, indicating the depth calculated through the model. $\left|T\right|$ is the number of elements in the test set.

(5) $$RMSELog=\sqrt{\frac{1}{\left|T\right|}\sum_{i=1}^{T}\left\|{logd}_{i}-logd_{i}^{*}\right\|}$$

(6) $$SqRel=\frac{1}{\left|T\right|}\sum_{i=1}^{T}\frac{{\left\|d_{i}-d_{i}^{*}\right\|}^{2}}{d_{i}^{*}}$$

In Equations (5) and (6), T also represents the total number of samples in the test set, i.e., the number of pixels in the actual depth map; $d_{i}^{*}$ denotes the actual depth value, while $d_{i}$ is the depth value predicted by the model. $\left|T\right|$ is the number of elements in the test set.

(7) $$\left\{\begin{array}{c} \delta_{1}=\max\left(\frac{d_{i}}{d_{i}^{*}},\frac{d_{i}^{*}}{d_{i}}\right)<1.25\\ \delta_{2}=\max\left(\frac{d_{i}}{d_{i}^{*}},\frac{d_{i}^{*}}{d_{i}}\right)<1.25^{2}\\ \delta_{3}=\max\left(\frac{d_{i}}{d_{i}^{*}},\frac{d_{i}^{*}}{d_{i}}\right)<1.25^{3} \end{array}\right.$$

In Equation (7), max(a,b) denotes the maximum value between a and b. $d_{i}^{*}$ represents the actual depth value, while $d_{i}$ is the depth value predicted by the model. $\delta_{1}$, $\delta_{2}$, and $\delta_{3}$ are used to evaluate the accuracy of the depth estimation model. $\delta_{1}$ measures whether the relative error between the predicted depth and the true depth is less than 1.25, indicating an error within 25%; $\delta_{2}$ checks if the error is less than 1.25^2^, tolerating a maximum error of 56.25%; $\delta_{3}$ permits a maximum error of 1.25^3^, corresponding to a maximum error of 95.31%. These thresholds help evaluate the model’s performance under different accuracy requirements, with smaller thresholds indicating more precise predictions.

The smaller the RMSE, RMSELog, AbsRel, and SqRel values calculated by the model, and the larger the accuracy δ, the better the model performs.

### 4.4. Loss Functions and Iterative Training

During training, to mitigate the influence of extreme values, the experiment employs a scale-invariant log loss function [15]. This function is motivated by the requirement for scale invariance in depth estimation, particularly addressing discrepancies between distant and near-range depth data. Standard loss functions may be overly sensitive to errors in distant data. The scale-invariant log loss mitigates this error influence by taking the logarithm of both the predicted and true depth maps, enabling the model to exhibit greater stability across different scales. This method is widely adopted in existing monocular depth estimation models. The calibration-invariant error is expressed by Equation (8).

(8) $$y_{i}=logx_{i}-logx_{i}^{*}$$

In Equation (8), $x_{i}$ denotes the predicted depth map, while $x_{i}^{*}$ represents the true depth map. By taking the logarithm of both the predicted and true depth maps and subtracting them, the region in the true depth map containing valid depth information for pixel N in the RGB image can be calculated [38]. Subsequently, due to the limitations of 3D sensors, depth data collected at close range is dense, while data at long range is sparse. To mitigate this imbalance, the loss function from [39] is adopted as the base loss function T to compare the predicted depth map with the true depth map, as shown in Equation (9).

(9) $$T\left(x_{i},x_{i}^{*}\right)=\frac{1}{N}\sum_{i}y_{i}^{2}-\frac{\mu }{N^{2}}{\left(\sum_{i}y_{i}\right)}^{2}$$

In Equation (9), the balance factor μ is set to 0.85, consistent with reference [15]. The variance penalty term reduces estimation errors for long-range depths by penalizing the variance of prediction errors, thereby enhancing model stability on long-range depth data. This approach originates from [15] and is implemented in [39], which proposes that variance penalties balance dense near-range data with sparse far-range data in depth datasets. This ensures the model does not neglect far-range depth estimation due to the excessive density of near-range data. This loss has been applied in multiple studies [15,17,38,39].

(10) $$L=\alpha \sqrt{T\left(x_{i},x_{i}^{*}\right)}$$

In Equation (10), L denotes the final loss function. The hyperparameter α is set to 10, consistent with Reference [15] and Reference [39].

During model training, the AdamW optimizer is employed. Due to the massive dataset size, training is time-consuming. To enhance efficiency while maintaining training effectiveness, selecting an appropriate learning rate strategy and number of epochs can help the model rapidly converge to its optimal values.

#### 4.4.1. The Impact of Total Training Epochs

During the training process of the EFDepth model, its loss value gradually decreased and stabilized as training progressed, indicating that the model was converging. To comprehensively evaluate model performance, we saved multiple training checkpoints for testing, including those from epochs 1 (Epoch 1), 5 (Epoch 5), 10 (Epoch 10), 15 (Epoch 15), 20 (Epoch 20), 25 (Epoch 25), 30 (Epoch 30), and 35 (Epoch 35). Testing was conducted across multiple epochs on the KITTI dataset, revealing that the model checkpoint at epoch 33 achieved optimal performance across all evaluation metrics. Consequently, we formally designate the training results from epoch 33 as the final EFDepth model.

**Figure 9:**
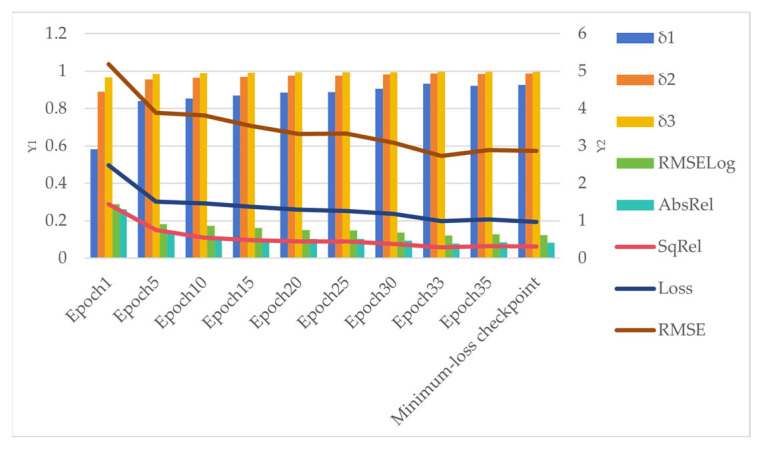
Changes in training epochs and metrics.

To investigate overfitting, we continued training beyond epoch 33 and obtained checkpoints with lower training losses (Minimum-loss checkpoint). Figure 9 illustrates the evolution of performance metrics from epoch 1 to epoch 35, alongside the final EFDepth model and the checkpoint with the minimum loss.

As shown in Figure 9, the bar chart uses the left Y1 axis as its vertical coordinate, while the line chart uses the right Y2 axis. Observation reveals that during the early training phase, both the model’s error metrics and loss values gradually decrease with increasing iterations, while accuracy metrics consistently increase. This indicates the model is in an effective learning phase. All metrics peak at the epoch 33 iteration, corresponding to our selected EFDepth model. However, starting from the 35th iteration, despite the training loss continuing to decrease, multiple performance metrics on the test set begin to deteriorate. The evaluation performance of the minimum loss checkpoint obtained from further training also fails to surpass that of the 33rd iteration. This clearly indicates that the model begins to overfit after the epoch 33 iteration.

Therefore, we set the total training epochs to 35. This setting represents a comprehensive trade-off between model performance and overfitting risk. Experiments show this configuration ensures sufficient model convergence while halting training early enough to avoid overfitting.

Experiments were conducted with a batch size of 8, and the full 35-epoch training cycle took approximately 90 h. Under this configuration, the finalized EFDepth model achieved an optimal performance equilibrium. Its EC-Net encoder and LapFA-Net decoder synergistically enhance predictive capabilities through multiscale feature optimization and contextual modeling, while ensuring stable convergence during training and high-quality depth map outputs.

#### 4.4.2. The Impact of Learning Rate

The choice of learning rate impacts training effectiveness, so three learning rate strategies were selected for training and analysis: dynamic learning rate (0.0001 → 0.00001), a fixed learning rate of 0.0001, and a fixed learning rate of 0.00001. The experimental results of the high-performing models trained using these three learning rates are shown in Table 1.

The dynamic learning rate adjustment strategy follows Reference [15], employing a polynomial decay approach to balance parameter exploration efficiency and convergence stability during model training. Specifically, the learning rate is initially set to a preset baseline rate (baselr) and gradually decays smoothly with training iterations, ultimately converging to a preset minimum learning rate (endlr). The adjustment formula follows a fast-then-slow decay pattern, as shown in Equation (11).

(11) lri=baselr−endlr×1−itotal0.5+endlr

In Equation (11), ${lr}_{i}$ denotes the learning rate for the i-th iteration, and total represents the total number of iterations. The training process varies with the selected learning rate.

Table 1 experimental results demonstrate that the dynamic learning rate model achieves optimal performance, while fixed learning rates of 0.0001 and 0.00001 exhibit inferior results. The core reason lies in the inability of fixed learning rates to adapt to the demands of the entire training epoch: the former, due to its fixed step size, struggles to perform fine-grained parameter optimization during the latter stages of training, often oscillating near the optimal solution and consequently yielding inferior accuracy and error metrics compared to the dynamic learning rate; The latter, with excessively small step sizes, not only converges slowly in the early training phase but also tends to get stuck in local minima, ultimately yielding the worst performance due to insufficient convergence. In contrast, the dynamic learning rate adapts to different training stages, facilitating rapid convergence in the early phase and enabling fine-tuning in the later phase. Therefore, a dynamic learning rate strategy is adopted during training.

### 4.5. Ablation Experiment

This paper adopts an encoder–decoder network architecture, where the base encoder is MobileNetV3 and the base decoder employs a five-layer Laplacian pyramid structure (termed Lap-Net). Building upon this foundation, the encoder EC-Net incorporates MobileNetV3-E and ETFBlock modules, while the decoder LapFA-Net utilizes a five-layer Laplacian pyramid structure with FMA modules integrated only in layers D1 to D4. To clarify the impact of adding the FMA module at layer D5, an ablation experiment was designed where the FMA module participates in every layer of the five-layer Laplacian pyramid structure. This scheme is termed Lap5FA-Net. Given that ETFBlock is an extended version of the ASPP, the ablation experiments includes a comparative analysis between the ASPP module and the ETFBlock. To evaluate the impact of each module on performance, multiple ablation experiments were conducted on a unified hardware platform using two datasets. Specific module configurations are detailed in Table 2, and ablation results are summarized in Table 3 and Table 4.

As shown in Table 2, Table 3 and Table 4, through stepwise analysis of ablation experiments, the specific contribution of each module to model performance becomes evident.

On the KITTI dataset and the NYU Depth V2 dataset, experimental results demonstrate that the integrated MobileNetV3-E, ETFBlock, and Laplacian pyramid structure with FMA module achieve optimal performance through synergistic collaboration, fully validating the functionality and improvement value of each component. When MobileNetV3-E is applied alone for basic feature extraction, its performance is the poorest due to the absence of subsequent processing modules. However, when combined with either ETFBlock or ASPP, the RMSE drops sharply, confirming that its feature optimization potential requires activation through collaborative modules. As an extended version of ASPP, ETFBlock cannot definitively claim superiority over any single module. Under identical configurations, experiments employing ETFBlock demonstrated lower root mean square error (RMSE) compared to ASPP. However, this advantage only materializes when ETFBlock collaborates with other modules, such as MobileNetV3-E. This demonstrates that while inheriting the multi-scale feature processing philosophy, the ETFBlock achieves more efficient feature refinement through structural optimization. Within this model, the ETFBlock proves more suitable than the ASPP module. In this model, ETFBlock demonstrates greater applicability than the ASPP module. However, both have their respective strengths and weaknesses, and neither holds an absolute advantage over the other. The Laplacian Pyramid architecture with the FMA module further enhances multi-scale feature fusion accuracy. Compared to EFDepth, Experiment 9 (without FMA) and Experiment 16 (five-layer FMA) both exhibit reduced root mean square error (RMSE). Thus, a four-layer FMA is sufficient and provides critical support for overall performance improvement. The EFDepth model demonstrated optimal performance across all metrics, outperforming any combination approach. This validated the applicability of MobileNetV3-E, ETFBlock, and LapFA-Net.

The progressive introduction of MobileNetV3-E, ETFBlock, and FMA modules has continuously enhanced model accuracy and stability. MobileNetV3-E synergizes with ETFBlock to strengthen feature extraction capabilities and multi-scale information fusion, thereby improving model performance. The FMA module, combined with the Laplacian pyramid structure, enhances the complementarity between features at different scales and the contextual modeling capability, ultimately improving depth estimation accuracy. Compared to using any single module alone, the synergistic application of all three yields superior depth map prediction results. The collaborative effect among the three modules optimizes the overall model performance, with the EFDepth configuration ultimately demonstrating the best accuracy and stability.

### 4.6. Comparative Experiment

To enhance the credibility of the model results, comparative experiments were conducted. Under identical experimental conditions, Lite-mono [11], Lapdepth [15], Hr-depth [23], Monodepth2 [24], and FCMdepth [25] were compared with the EFDepth model. These five models were evaluated on the dataset. To assess robustness, random noise was added to the data used for evaluating both datasets. In the experiments, the robustness test was named EFDepth-B. Through comparative experiments, we analyze performance across different datasets to better evaluate the strengths and weaknesses of the EFDepth model. The comparative results are shown in Table 5.

The comparative experimental results in Table 5 demonstrate that the EFDepth model exhibits certain advantages: it achieves optimal performance across three depth estimation error metrics, such as RMSE, SqRel, and others, while also yielding the highest accuracy metrics δ_1_, δ_2_, and δ_3_. Furthermore, this model requires fewer parameters and has lower FLOPs (Floating Point Operations). Even when noise is introduced, causing a slight decline in test performance, the results remain satisfactory. Consequently, EFDepth leads in error control and depth prediction accuracy, exhibiting good monocular depth estimation capabilities.

**Figure 10:**
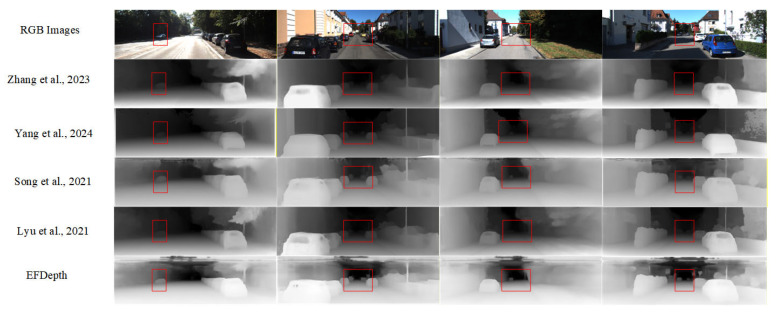
Display of predicted depth images. This method can clearly distinguish object details, as highlighted by the red bounding box. From top to bottom: Row 1: Input RGB image. Row 2: Prediction from Lite-Mono [11]. Row 3: Prediction from Depth Anything V2 [12]. Row 4: Prediction from LapDepth [15]. Row 5: Prediction from HR-Depth [23]. Row 6: Prediction from our proposed EEDepth model.

EFDepth demonstrates reduced error and improved accuracy across both datasets, validating its effectiveness and robustness. To further illustrate its reliability, depth map predictions were generated for selected images from KITTI dataset. The prediction results are shown in Figure 10. Compared to other models, EFDepth exhibits greater stability in depth prediction, particularly in scenes with intricate details (e.g., areas highlighted in red boxes), where its predictions are more accurate and closer to the true depth values.

Observing Figure 10, EFDepth demonstrates advantages in KITTI dataset scenes: on streets with dense trees or vehicles and high brightness (first column), it accurately predicts depth in highly reflective areas, clearly distinguishing vehicles from surrounding environments; on dimly lit streets (second column), the generated depth map remains stable and smooth overall; In ordinary street scenes (third column), it renders vehicle and building edges more sharply with clearer depth hierarchy; on shaded ordinary streets (fourth column), it captures distant vehicles with greater clarity, achieving more comprehensive depth prediction.

As shown in Table 5, the EFDepth model demonstrates limited improvement in some metrics but overall performance gains. Error metrics generally decrease while accuracy values like δ_1_ simultaneously increase, enhancing the prediction quality of depth maps. This advancement is visually evident, as demonstrated in Figure 10. The model delivers clearer and more coherent predictions along object edges and complex structures, reducing blurring and distortion. Consequently, EFDepth’s enhancement extends beyond quantitative metrics to strengthen depth information, offering practical value across various domains.

## 5. Conclusions

The proposed EFDepth model features a synergistic encoder–decoder architecture: the EC-Net encoder integrates MobileNetV3-E and ETFBlock to optimize features and expand the receptive field, while the LapFA-Net decoder employs a five-layer Laplacian pyramid structure combined with FMA modules to enable hierarchical depth prediction and enhanced contextual modeling. This model alleviates the core challenges of feature extraction and contextual modeling for depth estimation. Its enhanced depth prediction accuracy and detail recovery provide robust support for practical applications such as 3D reconstruction, autonomous driving, structural external crack damage detection, and building automation. For instance, in our self-developed structural external crack damage detection robot, integrating 3D vision technology enables precise identification of cracks and their progression trends. However, the model exhibits limitations in robustness under extreme scenarios, suggesting potential future research directions including multimodal data fusion and advanced attention mechanisms.

## Figures and Tables

**Table 1:** Training Metrics Variation with Learning Rate Selection.

Learning Rate	Loss	RMSE↓	RMSELog↓	AbsRel↓	SqRel↓	δ_1_↑	δ_2_↑	δ_3_↑
0.0001	1.093	2.927	0.130	0.088	0.344	0.917	0.984	0.996
0.00001	1.674	4.197	0.189	0.129	0.703	0.831	0.953	0.986
Dynamic learning rate	**0.986**	**2.734**	**0.120**	**0.079**	**0.294**	**0.932**	**0.988**	**0.997**

Note: **↓** indicates that lower values are preferable for this metric, while **↑** indicates that higher values are preferable. Bolded values represent the optimal values for the metrics in this column.

**Table 2:** Comparison Table for Module Combination Ablation Experiment Configuration.

Module Selection	MovbileNetV3	ASPP	Lap-Net	Lap5FA-Net	MovbileNetV3-E	ETFBlock	LapFA-Net
Experiment 1	√	×	√	×	×	×	×
Experiment 2	√	×	×	×	×	×	√
Experiment 3	√	×	√	×	×	√	×
Experiment 4	√	×	×	×	×	√	√
Experiment 5	√	√	×	×	×	×	√
Experiment 6	√	√	√	×	×	×	×
Experiment 7	×	×	√	×	√	×	×
Experiment 8	×	×	×	×	√	×	√
Experiment 9	×	×	√	×	√	√	×
Experiment 10	×	√	×	×	√	×	√
Experiment 11	×	√	√	×	√	×	×
Experiment 12	√	×	×	√	×	×	×
Experiment 13	√	×	×	√	×	√	×
Experiment 14	√	√	×	√	×	×	×
Experiment 15	×	×	×	√	√	×	×
Experiment 16	×	×	×	√	√	√	×
Experiment 17	×	√	×	√	√	×	×
EFDepth	×	×	×	×	√	√	√

Note: √ indicates adoption, × indicates non-adoption.

**Table 3:** Ablation Experiment Results on KITTI dataset.

Experiment Name	RMSE↓	RMSELog↓	AbsRel↓	SqRel↓	δ_1_↑	δ_2_↑	δ_3_↑
Experiment 1	5.878	0.254	0.168	1.206	0.762	0.904	0.962
Experiment 2	4.647	0.198	0.130	0.746	0.817	0.946	0.984
Experiment 3	3.046	0.137	0.093	0.380	0.907	0.982	0.995
Experiment 4	3.272	0.140	0.096	0.404	0.899	0.980	0.995
Experiment 5	3.206	0.139	0.095	0.392	0.900	0.981	0.996
Experiment 6	3.413	0.151	0.102	0.448	0.884	0.976	0.987
Experiment 7	16.656	0.908	1.099	23.989	0.121	0.257	0.482
Experiment 8	16.793	0.866	1.019	22.337	0.149	0.332	0.507
Experiment 9	3.037	0.134	0.090	0.360	0.911	0.983	0.996
Experiment 10	3.757	0.173	0.123	0.603	0.853	0.961	0.989
Experiment 11	2.939	0.130	0.088	0.343	0.914	0.984	0.996
Experiment 12	3.922	0.173	0.120	0.584	0.850	0.963	0.990
Experiment 13	3.042	0.133	0.089	0.363	0.912	0.983	0.996
Experiment 14	3.089	0.148	0.110	0.405	0.892	0.979	0.995
Experiment 15	18.051	1.013	1.067	25.568	0.123	0.283	0.416
Experiment 16	3.057	0.136	0.092	0.378	0.908	0.982	0.996
Experiment 17	3.463	0.159	0.112	0.525	0.873	0.971	0.992
EFDepth	**2.734**	**0.120**	**0.079**	**0.294**	**0.932**	**0.988**	**0.997**

Note: **↓** indicates that lower values are preferable for this metric, while **↑** indicates that higher values are preferable. Bolded values represent the optimal values for the metrics in this column.

**Table 4:** Ablation Experiment Results on NYU Depth V2 dataset.

Experiment Name	RMSE↓	AbsRel↓	SqRel↓	δ_1_↑	δ_2_↑	δ_3_↑
Experiment 1	0.604	0.137	0.171	0.831	0.966	0.990
Experiment 2	0.613	0.140	0.173	0.829	0.964	0.985
Experiment 3	0.533	0.136	0.173	0.838	0.970	0.990
Experiment 4	0.530	0.135	0.097	0.843	0.969	0.991
Experiment 5	0.525	0.136	0.091	0.867	0.974	0.993
Experiment 6	0.528	0.137	0.088	0.854	0.974	0.993
Experiment 7	0.897	0.196	0.274	0.596	0.851	0.939
Experiment 8	0.920	0.246	0.281	0.541	0.840	0.933
Experiment 9	0.407	0.111	0.052	0.896	0.977	0.994
Experiment 10	0.411	0.113	0.056	0.895	0.976	0.993
Experiment 11	0.541	0.135	0.082	0.837	0.965	0.989
Experiment 12	0.522	0.140	0.068	0.876	0.971	0.993
Experiment 13	0.514	0.139	0.069	0.883	0.976	0.994
Experiment 14	0.515	0.163	0.073	0.870	0.975	0.993
Experiment 15	0.647	0.178	0.192	0.685	0.973	0.954
Experiment 16	0.362	0.110	0.049	0.920	0.975	0.994
Experiment 17	0.515	0.141	0.070	0.873	0.972	0.993
EFDepth	**0.358**	**0.106**	**0.045**	**0.929**	**0.980**	**0.995**

Note: **↓** indicates that lower values are preferable for this metric, while **↑** indicates that higher values are preferable. Bolded values represent the optimal values for the metrics in this column.

**Table 5:** Comparative Experiment Results.

Datasets	Model Name	Parameter/M	FLOPs/G	RMSE↓	AbsRel↓	SqRel↓	δ_1_↑	δ_2_↑	δ_3_↑
KITTI	Lite-mono [11]	8.7	11.2	4.986	0.125	0.935	0.859	0.951	0.980
	Lapdepth [15]	73.5	36.5	3.995	0.096	0.552	0.892	0.972	0.992
	Hr-depth [23]	14.6	7.8	4.800	0.109	0.955	0.884	0.961	0.981
	Monodepth2 [24]	14.8	8.0	4.841	0.117	0.847	0.871	0.960	0.981
	FCMdepth [25]	5.5	1.2	3.164	0.099	0.407	0.896	0.980	0.995
	EFDepth-B (ours)	5.8	1.4	2.961	0.082	0.354	0.923	0.983	0.995
	EFDepth (ours)	5.8	1.4	**2.734**	**0.079**	**0.294**	**0.932**	**0.988**	**0.997**
NYU Depth V2	Lite-mono [11]	8.7	11.2	0.457	0.118	0.093	0.878	0.963	0.988
	Lapdepth [15]	73.5	36.5	0.439	0.111	0.055	0.881	0.968	0.992
	Hr-depth [23]	14.6	7.8	0.572	0.151	0.077	0.885	0.960	0.983
	Monodepth2 [24]	14.8	8.0	0.731	2.121	1.256	0.816	0.953	0.972
	FCMdepth [25]	5.5	1.2	0.367	0.101	0.047	0.892	0.975	0.991
	EFDepth-B (ours)	5.8	1.4	0.361	0.110	0.047	0.911	0.977	0.992
	EFDepth (ours)	5.8	1.4	**0.358**	**0.106**	**0.045**	**0.929**	**0.980**	**0.995**

Note: **↓** indicates that lower values are preferable for this metric, while **↑** indicates that higher values are preferable. Bolded values represent the optimal values for the metrics in this column.

## Data Availability

The data presented in this study are available in KITTI Visual Benchmark Dataset at https://www.cvlibs.net/datasets/kitti/index.php (accessed on 1 December 2025) and in the NYU Depth Dataset V2 at https://cs.nyu.edu/~fergus/datasets/nyu_depth_v2.html (accessed on 1 December 2025). These data were derived from the following resources available in the public domain: KITTI Visual Benchmark Dataset (https://www.cvlibs.net/datasets/kitti/index.php) and NYU Depth Dataset V2 (https://cs.nyu.edu/~fergus/datasets/nyu_depth_v2.html).

## Abbreviations

The following abbreviations are used in this manuscript:

MDE	Monocular Depth Estimation
GPU	Graphics Processing Unit
CUDA	Compute Unified Device Architecture
RGB	Red Green Blue
ASPP	Atrous Spatial Pyramid Pooling
CNN	Convolutional Neural Networks
FEM	Feature Enhancement Module
FMA	Fourier Modulated Attention
RMSE	Root Mean Square Error
RMSELog	Root Mean Square Logarithmic Error
AbsRel	Absolute Relative Error
SqRel	Square Relative Error
3D	Three Dimensional
FLOPs	Floating Point Operations

## References

[1] Ming Y., Meng X., Fan C., Yu H. (2021). Deep learning for monocular depth estimation: A review. Neurocomputing.

[2] Patni S., Agarwal A., Arora C. Ecodepth: Effective conditioning of diffusion models for monocular depth estimation. Proceedings of the IEEE/CVF Conference on Computer Vision and Pattern Recognition.

[3] Gurram A., Tuna A.F., Shen F., Urfalioglu O., López A.M. (2021). Monocular depth estimation through virtual-world supervision and real-world sfm self-supervision. IEEE Trans. Intell. Transp. Syst..

[4] Yao Y., Luo Z., Li S., Fang T., Quan L. Mvsnet: Depth inference for unstructured multi-view stereo. Proceedings of the European Conference on Computer Vision (ECCV).

[5] Zhao G., Wei H., He H. (2025). IAFMVS: Iterative Depth Estimation with Adaptive Features for Multi-View Stereo. Neurocomputing.

[6] Guizilini V., Lee K.H., Ambruş R., Gaidon A. (2022). Learning optical flow, depth, and scene flow without real-world labels. IEEE Robot. Autom. Lett..

[7] Long X., Zheng Y., Zheng Y., Tian B., Lin C., Liu L., Zhao H., Zhou G., Wang W. (2024). Adaptive surface normal constraint for geometric estimation from monocular images. IEEE Trans. Pattern Anal. Mach. Intell..

[8] Long X., Liu L., Li W., Theobalt C., Wang W. Multi-view depth estimation using epipolar spatio-temporal networks. Proceedings of the IEEE/CVF Conference on Computer Vision and Pattern Recognition.

[9] Masoumian A., Rashwan H.A., Cristiano J., Asif M.S., Puig D. (2021). Monocular Depth Estimation Using Deep Learning: A Review. Sensors.

[10] Dong X., Garratt M.A., Anavatti S.G., Abbass H.A. (2022). Towards real-time monocular depth estimation for robotics: A survey. IEEE Trans. Intell. Transp. Syst..

[11] Zhang N., Nex F., Vosselman G., Kerle N. Lite-mono: A lightweight cnn and transformer architecture for self-supervised monocular depth estimation. Proceedings of the IEEE/CVF Conference on Computer Vision and Pattern Recognition.

[12] Yang L., Kang B., Huang Z., Zhao Z., Xu X., Feng J., Zhao H. (2024). Depth anything v2. Adv. Neural Inf. Process. Syst..

[13] Song J., Lee S.J. (2025). RGB-Based Visual–Inertial Odometry via Knowledge Distillation from Self-Supervised Depth Estimation with Foundation Models. Sensors.

[14] Hu H., Feng Y., Li D., Zhang S., Zhao H. (2024). Monocular Depth Estimation via Self-Supervised Self-Distillation. Sensors.

[15] Song M., Lim S., Kim W. (2021). Monocular depth estimation using laplacian pyramid-based depth residuals. IEEE Trans. Circuits Syst. Video Technol..

[16] Zhu Y., Ren R., Dong W., Li X., Shi G. (2024). TSUDepth: Exploring temporal symmetry-based uncertainty for unsupervised monocular depth estimation. Neurocomputing.

[17] Lu J., Huang T., Li P., Dou Z., Lin C., Cui Z., Dong Z., Yeung S.K., Wang W., Liu Y. Align3r: Aligned monocular depth estimation for dynamic videos. Proceedings of the Computer Vision and Pattern Recognition Conference.

[18] Yang W.-J., Wu C.-C., Yang J.-F. (2025). Residual Vision Transformer and Adaptive Fusion Autoencoders for Monocular Depth Estimation. Sensors.

[19] Hwang S.J., Park S.J., Baek J.H., Kim B. (2022). Self-supervised monocular depth estimation using hybrid transformer encoder. IEEE Sens. J..

[20] Li Z., Wang X., Liu X., Jiang J. (2024). BinsFormer: Revisiting Adaptive Bins for Monocular Depth Estimation. IEEE Trans. Image Process..

[21] Zhao C., Zhang Y., Poggi M., Tosi F., Guo X., Zhu Z., Huang G., Tang Y., Mattoccia S. Monovit: Self-supervised monocular depth estimation with a vision transformer. Proceedings of the 2022 international conference on 3D vision (3DV).

[22] Li Z., Chen Z., Liu X., Jiang J. (2023). Depthformer: Exploiting long-range correlation and local information for accurate monocular depth estimation. Mach. Intell. Res..

[23] Lyu X., Liu L., Wang M., Kong X., Liu L., Liu Y., Chen X., Yuan Y. Hr-depth: High resolution self-supervised monocular depth estimation. Proceedings of the AAAI Conference on Artificial Intelligence.

[24] Godard C., Mac Aodha O., Firman M., Brostow G.J. Digging into self-supervised monocular depth estimation. Proceedings of the IEEE/CVF International Conference on Computer Vision.

[25] Liu F., Shao X., Zhang C., Wang L., Ren J. (2025). FCMdepth: Monocular depth estimation framework with multi-scale feature optimization. J. Comput. Appl..

[26] Wang P., Liu S., Li Q., Yi Y., Wang J. (2025). MG-Mono: A lightweight multi-granularity method for self-supervised monocular depth estimation. Pattern Recognit..

[27] Lopez J., Vargas E., Arguello H. (2024). Depth estimation from a single optical encoded image using a learned colored-coded aperture. IEEE Trans. Comput. Imaging.

[28] Das D., Das A.D., Sadaf F. (2025). Enhanced encoder–decoder architecture for accurate monocular depth estimation. Vis. Comput..

[29] Howard A., Sandler M., Chu G., Chen L.C., Chen B., Tan M., Wang W., Zhu Y., Pang R., Vasudevan V. Searching for mobilenetv3. Proceedings of the IEEE/CVF International Conference on Computer Vision.

[30] Tian X., He Z., Zhang Y., Liu F., Gu T. (2025). MsGf: A Lightweight Self-Supervised Monocular Depth Estimation Framework with Multi-Scale Feature Extraction. Sensors.

[31] Olmo J.J.L.D., Ballesteros E.P., Gómez Á.L.P., López-de-Teruel P.E., Ruiz A., Clemente F.J.G. (2025). Fog computing-driven logistics: Leveraging few-shot learning and foundational computer vision models. Clust. Comput..

[32] Liu X., Tang S., Feng M., Guo X., Zhang Y., Wang Y. (2025). A simple monocular depth estimation network for balancing complexity and accuracy. Sci. Rep..

[33] Aggarwal S., Bhangale T., Paul A., Bharambe S., More J. Face Recognition and Navigation Aid for the Visually Impaired. Proceedings of the 2025 International Conference on Emerging Trends in Industry 4.0 Technologies (ICETI4T).

[34] Zhang Y., Ye M., Zhu G., Liu Y., Guo P., Yan J. (2024). FFCA-YOLO for Small Object Detection in Remote Sensing Images. IEEE Trans. Geosci. Remote Sens..

[35] Lo C.C., Vandewalle P. (2024). Expanding sparse radar depth based on joint bilateral filter for radar-guided monocular depth estimation. Sensors.

[36] Li F., Cong R., Wu J., Bai H., Wang M., Zhao Y. (2025). Srconvnet: A transformer-style convnet for lightweight image super-resolution. Int. J. Comput. Vis..

[37] Song X., Tan Y., Ning J., Lu X., Hei X. (2025). Unsupervised Monocular Depth Estimation Method Based on Multimodal Interaction Perception. IEEE Sens. J..

[38] Ljunggren O. (2025). Monocular Depth Estimation for Satellite Depth Map Enhancement. Master’s Thesis.

[39] Son E., Choi J., Song J., Jin Y., Lee S.J. (2023). Monocular depth estimation from a fisheye camera based on knowledge distillation. Sensors.

